# Factors influencing drop-out of households from community based health insurance membership in rural districts of Gurage Zone, Southern Ethiopia: Community based case-control study

**DOI:** 10.3389/fpubh.2022.925309

**Published:** 2022-10-05

**Authors:** Kebebush Zepre, Fedila Yassin, Betelhem Tadesse, Omega Tolossa, Derbachew Hailemariam, Asegedech Wondimu, Fisha Alebel GebreEyesus, Tadesse Tsehay, Kenzudin Assfa

**Affiliations:** ^1^Department of Public Health, College of Medicine and Health Science, Wolkite University, Wolkite, Ethiopia; ^2^Department of Nursing, College of Medicine and Health Science, Wolkite University, Wolkite, Ethiopia

**Keywords:** CBHI, dropout, influencing, factors, Gurage Zone

## Abstract

**Background:**

Financial risk-sharing through community-based health insurance is a critical component of universal health coverage. However, its development is a great challenge, not only due to low enrollment but also due to the high dropout rate of members from the program, which threatens its sustainability. So far, the few existing studies in this area have focused on household enrollment into community-based health insurance, rather than on the number of members dropping out. This study aims to identify factors influencing households to drop out of community-based health insurance membership in rural districts of the Gurage Zone, Southern Ethiopia.

**Methods:**

A community-based case-control study was carried out from May to July 2021. Supplemented by qualitative focus group discussions. Multi-stage sampling was employed. An interviewer-administered prearranged tool was used for collecting data. Epi-data version 3.1 and SPSS version 21 were used for data entry and analysis. The association between factor and outcome variable was determined using binary logistic regression analysis at *p* < 0.05 and 95% CI. Qualitative data were analyzed thematically and triangulated.

**Results:**

From 525 (175 cases and 350 controls) rural household heads 171 cases and 342 controls responded, yielding a response rate of 97.7%. Of those, 73.1 and 69.0% were males in cases and controls, respectively. The statistically significant influencing factors associated with dropout from community-based health insurance were: highest wealth status (adjusted odds ratio [AOR] = 2.36, 95% confidence interval [CI]:1.14–4.87), unfavorable attitude toward CBHI (AOR: 1.81, 95% CI: 1.87–3.37), no illness experienced in the last 3 months (AOR: 5.21, 95% CI: 2.90–9.33). no frequent health facility visits (AOR:5.03, 95% CI:1.17–23.43), no exposure to indigenous community insurance (AOR:0.10, 95% CI: 0.03–0.37), not graduated in the model household (AOR: 3.20, 95% CI:1.75–5.83), being a member in the program for more than 3 years (AOR:0.55, 95% CI: 0.29–0.94), not trusting governing bodies (AOR:10.52, 95% CI:4.70–23.53), the ordered drug was not available in the contractual facility (AOR:14.62, 95% CI:5.37–39.83), waiting time was >3 h (AOR:4.26, 95% CI:1.70–10.66), and poor perception of service quality (AOR:12.38, 95%CI:2.46–62.24).

**Conclusion:**

The findings of this study illustrated various factors which positively and negatively influenced households to drop out from CBHI: wealth status, attitude toward CBHI, perceived poor provider attitude toward CBHI members, illness experience in the household, the experience of frequent health facility visits, model household graduation status, trust on CBHI committee (governing bodies), availability of a prescribed drug in the contractual health facility, waiting time and perceived quality of health service from the contractual facility, exposure to any of the indigenous insurance (IDIR and/or IQUB) and length of membership in program. We strongly recommend all responsible stakeholders give strong attention to promoting the community, and for providers to project a favorable attitude toward community-based health insurance, to achieve model household graduation, and improve quality of service by addressing the basic quality-related areas like waiting time, and drug availability).

## Introduction

How to pay for health services is a basic issue that must be addressed. Searching for a way to raise adequate resources is noticeably vital. Even though the major dependence of financial sources for the health system is on public funding, it is vital to guarantee access to healthcare while, at the same time, protecting families from catastrophic medical bills (1).

The healthcare financing issue is a major concern in low and middle-income countries (LMIC) where more than half of the people live under the poverty line. In these countries, the primary mode of payment for health services is direct out-of-pocket payment. Sadly, that limits those unable to pay direct out-of-pocket costs from accessing quality care (2). Since illness is unpredictable, the rural poor borrow money and/or sell their assets to cover healthcare expenses during an emergency. Moreover, it can considerably push entire households into poverty (2).

Community-based health insurance (CBHI) is a not-for-profit type of health insurance in which members pay a small amount of annual income (230–350 ETB in the Ethiopian context). It helps them to protect themselves from catastrophic expenses at the time of unpredictable illness. It covers the poor, the unemployed, and mostly those living in rural areas. Its enrollment is based on the voluntary participation of households (HHS). It lessens the equity gap between the haves and the have-nots in seeking healthcare (3). In many nations, including developing countries, CBHI has been considered the best approach to address the health access needs of all people to get the health services they need, when and where they need them, without worrying about payment at the service point (4).

The government of Ethiopia introduced CBHI in the year 2003 in 13 districts of the four regions with large populations (Oromia, Amhara, SNNP, and Tigray) as a pilot. After evaluating the feasibility and accomplishment of the program in the pilot districts, the government expanded its implementation to other districts. Currently, about 512 districts have implemented it; the aim is to enable the poor to get quality health services regardless of their economic status (5, 6). In the Gurage Zone, it was first implemented in 2003 EC. Currently, all districts of the Gurage Zone are implementing the program (6).

A household is considered a dropout from the CBHI program when they have not renewed their membership year after year. Although CBHI hopes to solve the hardship of healthcare expenses that confronts the poor, in LMIC its development is a proven challenge (7–10). Therefore, it is time to innovate various strategies to encourage all households to remain in the program. Worldwide, annually, about 44 million households are exposed to disastrous healthcare-related expenses, while about 25 million households are pushed into poverty due to direct out-of-pocket payments for healthcare services. The majority (>90 %) of these happen in LMICs (7). In Ethiopia, there is a high occurrence of morbidity and mortality due to health conditions that could be prevented and cured easily, such as malaria, pneumonia, and other respiratory illnesses. However, the community does not use the modern health service even if they are in need due to user fee charges at service points (11, 12).

In LMIC, CBHI shows the potential path to protect the poor from healthcare expense-related catastrophe and is one way to address the health access needs of the community (1, 8, 13). In Ethiopia, the Federal Ministry of Health (FMOH) has tried to implement various initiatives on health since 1993 to improve healthcare access and quality at the community level (health facility constriction, human resource development, health extension programs, and others), however none of them tackle the finance-related hardship (5). As a result, the government has committed to protect the poor from healthcare-related financial risks and launched CBHI in 2011 GC as a solution to alleviate these financial difficulties, and it has been introduced as one of the strategies to implement the district transformation agenda in the health sector transformation plan (HSTP) (11, 12).

However, the development of the CBHI program faces a great challenge that is not only due to low enrollment but also due to the high dropout rate, which causes CBHI to be unsustainable by decreasing its coverage directly. It also negatively affects further enrollment and dropout of HHS to and from the program (13–16). A study done in Ethiopia documented that enrollment a year after initiation increased only from 41 to 48%. On the other hand, 18 % of those who joined the program in the 1st year did not renew their membership the year after (15). Correspondingly, in our study area, even though there has been a new enrollee every year, the coverage of CBHI membership has not moved up. The HHS membership rate of the program is very low in this study area compared to the national expectation (68 vs. 85%)(6, 12).

The few existing studies focused on HHS' willingness to pay and enroll in CBHI. Yet, there is a paucity of evidence on what hinders HHS to renew membership year after year. Thus, we aimed to identify factors influencing the dropout of HHS from CBHI membership in CBHI implementing rural districts of the Gurage Zone, southern Ethiopia. The findings from this study will help government and health policymakers by providing evidence to guide strategy for proper interventions, help insurance agencies and health planners by providing inputs and direction to determine the basic parameters in scale-up districts, such as the amount of contribution and institutional arrangement, and help researchers to use this data as an input for further study.

## Methods and materials

### Study area and period

This study was conducted in selected districts of the Gurage Zone from May to July 2021. Gurage Zone is one of the administrative zones of the SNNPRs Region. It is located 155 km southwest of Addis Ababa. According to 2019 estimates, the total population of the Gurage Zone is about 1.8 million, of which more than 90% live in the rural part. Currently, it has a total of 16 districts and five town administrations, all of these implementing CBHI. Among these, only 10 of the rural districts were implementing CBHI before the year 2012 EC. Currently, there are six public hospitals, 67 health centers, 412 rural health posts, 24 medium, and 83 primary private clinics (6).

### Study design and population

A population-based case-control study design was used and supplemented by qualitative focus group discussions (FGDs). All rural households in CBHI implementing districts of the Gurage Zone, who registered for CBHI in the year 2012 EC or before, and either have not renewed their membership (for cases) or who have renewed their membership (for controls), and who have volunteered to take part in the study were considered as study participants. However, household heads, too sick to give consent or to be interviewed were disqualified.

### Sample size determination

The number of participants was calculated by considering CBHI-non-members (dropouts) as cases and CBHI members as control groups and using Epi-info version 7 following the supposition such as percentage of trained HHS, proportion of households perception toward health care quality, and educational status as exposure variables — predictors of dropout from CBHI membership — from a study conducted in Ghana and Tanzania (17, 18), with the supposition percentage outcome, power of 80%, ratio (case: Control) 1:2, 95% CI, Odds ratio, non-response rate (10 %) and design effect (1.5). Finally, take trained HH head as the key influencing factors for CBHI dropout, 8.05 % among cases and 20.28 % among controls, an Odds ratio of 2.15 which gives the largest sample size, 525. For qualitative focus group discussions (FGDs) by considering homogeneity of population, convenience for moderation, and cost, a total of 8 FGDs each consisting of 12 discussants were planned but based on information saturation, six FGDs were undertaken.

### Sampling procedure

**Figure 1:**
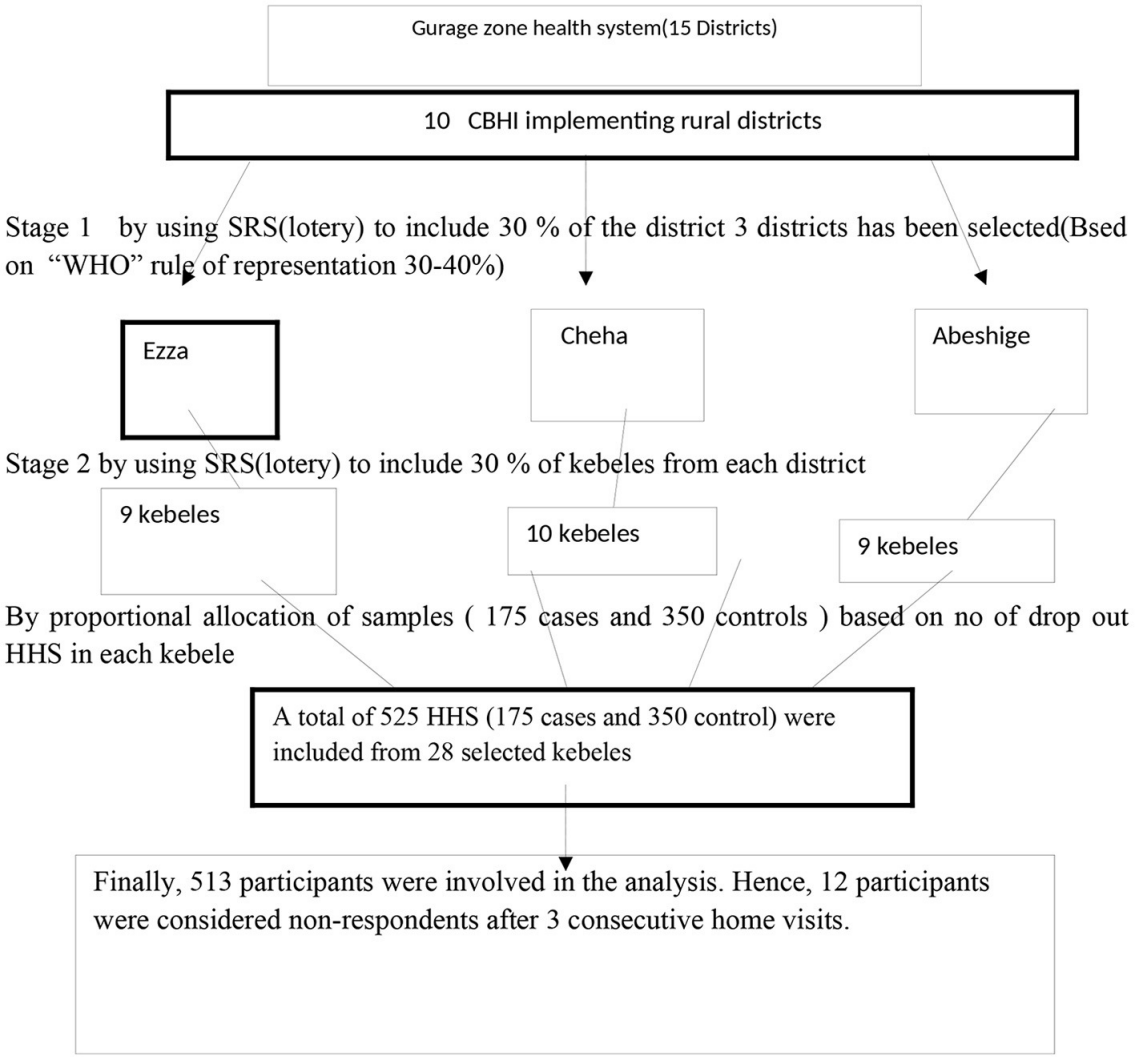
Schematic presentation of the sampling procedure for a study on factors influencing dropout of households from community-based health insurance, that clarifies the sample enrolled and dropout in each phase in southern Ethiopia, 2021.

A multi-stage sampling technique was deployed. Three districts (30% of the 10 CBHI implementing districts) (Ezza, Cheha, and Abeshige) were selected randomly using the lottery method and 30% of kebeles (wards) from each selected district (a total of 28 kebeles) were also selected, randomly by simple random sampling (SRS) (Based on the World Health Organization's (WHO's) rule of representation 30–40%). The sampling frame was obtained from the CBHI office of each district and/or family folders of each kebele. Finally, both cases and controls were selected randomly, by SRS. The sample was allocated per the total number of households (HHS) in each kebele independently (See Figure 1).

For the qualitative study, the purposive sampling technique was used for FGDs, with health professionals of each district, insurance management team leaders, health extension workers, leaders of health development armies of the community, and community members (from both CBHI members and dropped out HHS) not involved in the survey were considered based on their knowledge regarding CBHI and/or because they had a specific role in efforts to improve CBHI sustainability. A total of six FGDs were conducted, and each group consisted of 12 individuals (42 male and 30 female).

### Data collection tool and procedure

The tool was adapted and modified to the local context based on reviewed literature. It was prepared initially in English and then translated into local Amharic and back to English to ensure its reliability. The tool was used to gather data on the Socio-demographic and Socio-economic status of HHS, Individual HHH awareness and attitude toward CBHI, HH level variables, health services, and CBHI program-related variables.

By using an interviewer-administered structured questionnaire, a house-to-house survey of randomly selected households was carried out by 12 trained diploma nurses and three supervisors (BSc, PH). Overall supervision was undertaken by the principal investigator. 2 days of training were prearranged for data collectors and supervisors to understand the aim of the study, data collection tools, ethical issues, and procedures.

FGDs, with health professionals, insurance management team leaders, health extension workers, leaders of health development armies of the community, and community members (from both member and non-member HHS) from each selected district were used to explore the experience of the community regarding CBHI and their reasons for why households drop out from CBHI membership by using an open-ended questionnaire. FGDs were moderated by experts (MPH in health education) and note-takers, note-taking, and tape recording were used.

### Operational definitions

Awareness of insured and non-insured (dropout) HHS about CBHIS was measured by seven awareness questions, each score one. Good awareness is categorized as a value greater than or equal to the mean value and poor awareness as a value less than the mean value (7). The attitude of participants toward CBHIS was measured by a 5-point Likert scale score having 10 items. Finally, the individual sample mean score was computed and those who scored sample mean and above were judged as having favorable attitudes and those who scored less than sample means as having an unfavorable attitude toward CBHI (7).

HHS who joined the CBHI program in 2012 E.C or before and did not renew their program card in 2013 E.C were considered as cases (dropout). Households who joined the CBHI program in 2012 or before and renewed their program card in 2013 EC and who have an updated membership card on hand were considered as controls.

Household wealth status is household living status which has been constructed using HH asset data composed of different indicators and was adapted from EDHS 2016 (19) and modified to local and rural household contexts by incorporating agricultural products locally produced. It was measured by using information like the type of floor, roof, wall, water source, latrine, ownership of radio, bicycle, motorcycle, amount of grain (collected in the last production year), number of livestock, and ownership of farmland. After conducting a principal component analysis (PCA) by SPSS, the household's wealth was grouped in quintiles (from lowest to highest). The quintiles were Q1 (lowest), Q2 (2nd wealth quintile), Q3 (medium), Q4 (4th wealth quintile), and Q5 (highest).

### Data processing and analysis

Data was entered into Epi-data version 3.1 and exported to Statistical Package for Social Science (SPSS) version 21 for cleaning and analysis. Descriptive statistics were performed and findings were presented with text, tables, and figures. A principal component analysis was done using possessions of household assets to construct a wealth index as a proxy measure of household socioeconomic status. Assumptions of principal component analysis were checked. Accordingly, households were categorized into five wealth quintiles for further analysis.

Binary logistic regression was used to assess the association between each independent variable with the dependent variable. Model fitness was tested using Hosmer and Lemeshow statistics and (*p*-value > 0.05 showed a good fit model). All variables with *P* < 0.25 in the bivariate analysis were included in the final model of multivariable analysis to control all possible confounders. Numerical and graphical methods were used to test normality. In the Q-Q plot test of normality, the data point was close to the diagonal line, the data were normally distributed. An approximate bell-curve shape was observed in the histogram that the data may have come from a normal population. As well, the value of the Shapiro-Wilk Test was >0.05, so the data are normal. Multicollinearity was checked and variables with VIF above 10 and significant correlation were excluded from the model. Multicollinearity and interaction effects were analyzed and we did not find a significant effect modification among factors in the final model. The adjusted odds ratio along with 95% CI that was undertaken to identify independent factors of dropout from CBHI. *P*-value < 0.05 was considered to declare a result as a statistically significant association. The qualitative data were analyzed thematically and triangulated, by categorizing under the following sections: what is known about CBHI, the common social support mechanism in the community related to the financial issues for healthcare costs, which types of HH are more sustainable in CBHI membership, and what needs to improve in the future.

### Data quality management

To guarantee data quality, all individuals responsible for data collection and supervision were trained. The tool was pretested in one of the districts on 5% of total sample households to check for consistency, clarity, and sequence of questions, and also to familiarize the data collectors with the tool. Data were checked for completeness, accuracy, and consistency on a daily basis and then all necessary corrections were made. Data were entered and cleaned using Epi-data 3.1 before exporting to SPSS for analysis.

### Ethical consideration

Permission for the study was obtained from the Institutional Review Board (IRB) of Wolkite University, College of Medicine and Health Science. A permission letter was obtained from the Gurage Zone health department and the respective districts. Respondents' privacy was maintained by informing them that their names and personal identifiers would not be written on the questionnaire. The purpose of the study and the details of the consent process were well-explained to respondents in their preferred language. Finally, written informed consent was obtained from each respondent before data collection (all participants who showed their willingness to participate put their signature on the paper (even those who were not able to read and write put their signature by fingerprint). Respondents had been told that they have full rights to participate or refuse participation in the study and the right to stop at any time if not feeling at ease. The consent process was approved by the IRB of Wolkite University, College of Medicine and Health Science.

## Results

### Socio-demographic and economic characteristics of cases and controls

Of 525 (175 cases and 350 controls) rural household heads 171 cases and 342 controls responded, yielding a response rate of 97.7%. The majority of cases were more likely than controls to be male 73 vs. 69%, able to read and write 57.3 vs. 55.8%, and farmers 73.7 vs. 68.4%) (Table 2). Nearly half of the cases and controls were at a mean age of (48.2vs. 46.9 years), orthodox 54.5 vs. 55% (Table 1), and regarding educational level, completed primary education 33.3 vs. 33.3%. About 89.5% of cases and 95.9% of controls were from Gurage Ethnic group and about 84.8% of cases and 89.5% of controls were married (Table 1). Regarding respondents' wealth status, about 25.6% of cases and 22.9% of controls were from middle and 4th wealth quintiles correspondingly (Table 1).

**Table 1:** Socio-demographic and economic characteristics of the cases and controls of factors influencing dropout of HH from Community-based Health Insurance membership in rural districts of the Gurage Zone, southwest Ethiopia, 2021 (*n* = 513).

**Characteristics**	**Category**	**Cases = 171**	**Controls = 342**
		**Count (%)**	**Count (%)**
Sex	Male	**125(73.1)**	**236(69.0)**
	Female	46(26.9)	106(31.0)
Age	18-34	17(9.9)	32(9.4)
	35-64	142(83.0)	284(83.0)
	≥65	12(7.0)	26(7.6)
Religion	Orthodox	**93(54.4)**	**188(55)**
	Muslim	53(31.0)	124(36.3)
	Protestant	15(8.8)	24(7.0)
	Catholic	10(5.8)	6(1.8)
Ethnicity	Gurage	**154(89.5)**	**328(95.9)**
	Amhara	14(8.2)	7(2.0)
	Kebena	3(1.8)	4(1.2)
	Oromo	1(0.6)	3(0.9)
Marital status	Married	**145(84.8)**	**306(89.4)**
	Widowed	19(11.1)	33(9.6)
	Not married	7(4.1)	3(0.9)
Educational status	Can't read & write	73(42.7)	148(43.3)
	Read & write only	27(15.8)	51(14.9)
	Primary education(1-8)	57(33.3)	114(33.3)
	Secondary and above	14(8.2)	29(8.5)
Household headship	Husband	**124(72.5)**	**232(67.8)**
	Spouse	46(26.9)	110(32.2)
	Other	1(0.6)	0(0.0)
Occupation	Farmer	***126(73.7)***	***234(68.4)***
	Housewife	41(24.0)	102(29.8)
	Others	4(2.3)	6(1.8 %)
Wealth in quintiles	Lowest wealth quintile	29(17.2)	73(21.3)
	2^nd^ wealth quintile	40(23.4)	63(18.3)
	Middle wealth quintile	**44(25.6)**	60(17.6)
	4^th^ wealth quintile	24(13.8)	**78(22.9)**
	Highest wealth quintile	34(20.0)	68(19.9)
Mean age of the respondent		48.2 ±10.9 SD(R, 25-75)	46.9 ± 11.2 SD (R, 18-75)

SD, standard deviation; R, Range. Bold values are category with largest composition.

### Household-related characteristics of the cases and controls

Out of the total respondents, about 38% of case HHS and 48.5 % of control HHS has a family size of greater than five (large family size). About 32.7% of cases and 46.8% of control reported there is at least one child under 5 years old in their HH. Concerning the health status of the case and control, nearly half (48.5%) and 79.8%, respectively reported their family's health status as good while 3.5 and 1.2%, respectively reported it as poor (Table 2). Regarding the history of chronic illness in the household, 28.7% of cases and 33.0% of controls reported a family member with chronic illness. Regarding exposure to indigenous community insurance (IQUB, IDIR), 76.6% of cases and 90.4% of controls reported they were a member of an indigenous community insurance. Concerning being a model HH, less than half (45.6%) of cases and more than two third (67.5%) of controls responded that they graduated as model HH (Table 2).

**Table 2:** Household-related characteristics of the cases and controls of factors influencing dropout of HH from CBHI membership in rural districts of the Gurage Zone, southern Ethiopia, 2021 (*n* = 513).

**Variables**	**Category**	**Cases** = **171**		**Controls** = **342**	
		**Frequency (%)**		**Frequency (%)**	
Family size	< 5	106	62.0	176	51.5
	>5	65	38.0	166	48.5
Any < 5 years of age children in HH	Yes	56	32.7	160	46.8
	No	115	67.3	182	53.2
Health status (HHH self-reported) of the HH	Good	83	48.5	273	79.8
	Medium	82	48.0	65	19.0
	Poor	6	3.5	4	1.2
HH illness experience in the last 3 months	Yes	74	43.3	279	81.6
	No	97	56.7	63	18.4
Any member in HH with chronic illness	Yes	49	28.7	116	33.9
	No	122	71.3	226	66.1
Exposure to indigenous community insurances (IQUB, IDIR)	Yes	131	76.6	309	90.4
	No	40	23.4	33	9.6
HH model status (graduated by HEW as model family or not)	Yes	78	45.6	231	67.5
	No	93	54.4	111	22.5

HH, household; HEW, health extension worker.

### Awareness and attitudes of cases and controls toward CBHI

**Figure 2:**
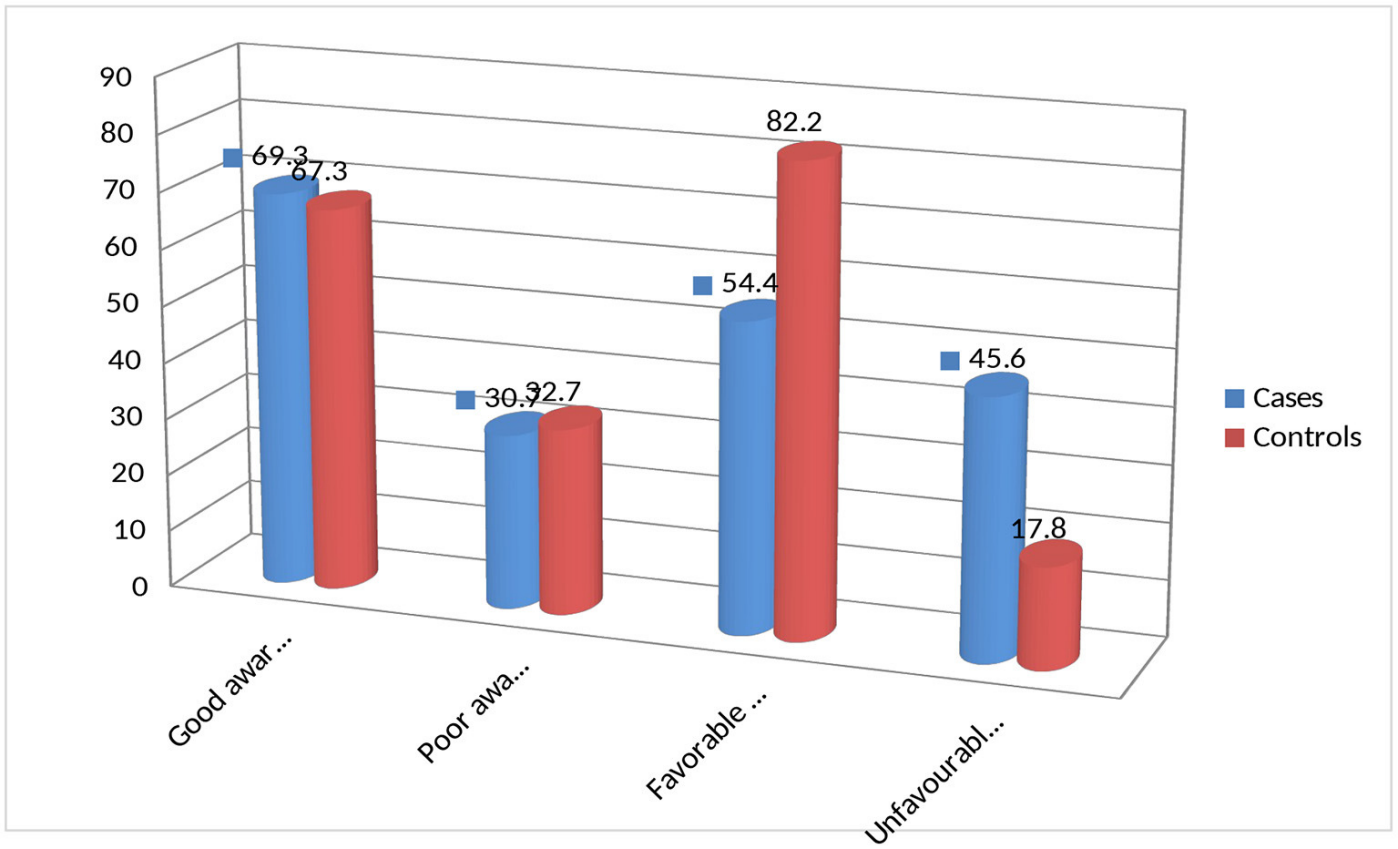
Awareness and attitudes of rural household heads toward CBHI as factors influencing dropout of HH from CBHI in rural districts of Gurage Zone, southwest Ethiopia, 2021.

Although almost all (99.4 % of cases and 100% of control) HH heads reported they had heard about CBHI, only 69.6% of cases and 67.3% of controls considered CBHI as prepayment for healthcare and sharing financial risk among members. Two-thirds, 66% of cases and 67% of controls, know the range of benefits package, and almost all case and control HHH know the amount and timing of premium payments. While 17% of cases and 1.8% of control HHH considered CBHI as paying tax to the government, 12.3% of cases and 31% of controls considered it as free health delivery by the government. Generally, 69.6% of cases and 67.3% of controls score mean and above and were considered as having a good awareness of CBHI (Figure 2). More than half (54.4%) of cases and 82.2% of controls had a favorable attitude toward CBHI (see Figure 2).

### Health service utilization and CBHI program-related characteristics of the cases and controls

About 36.8% of cases and 65.5% of controls stayed enrolled for 3 or more years. Regarding affordability of annual premium, 63.2% of cases and 87.1% of controls perceived the annual premium as affordable. Regarding government subsidy, only 0.6% of cases reported their premium covered by the government vs. 3.9% of control. Concerning trust in governing bodies, about 48% of cases and 93.6% of controls reported they have trust. Of the total respondents, 87.3% of cases and 83.0% of controls access the health facility within an hour's walk. About 81.9%) of cases and 100% of controls had visited the healthcare facilities in the last 3 months. Among those who have received service 65.0% of cases and 96.5% of controls reported the ordered drug available while 30.0% of cases and 3.5% of controls reported the ordered drug not available.

Out of 140 cases who have received service from the contractual health facility, about 68.6, 25.7, and 5.7% rated their satisfaction with existing CBHI services as not satisfied, satisfied, and neutral, respectively while ratings of 2.3, 40.1, and 57.6%, respectively were registered among controls. Generally, about 1.4% of cases and 24.8% of controls perceived the service quality as good while 98.6% of cases and 75.2% of controls perceived it as poor.

Respondents put their main reason for dropout from membership as follows (multiple responses): Ordered drugs not available at the facility and not reimbursed 121 (70.8%), service quality poor 89 (52%), service partiality between members and non-members 64 (37.4%), annual premium high 58 (33.9%), benefit package narrow 39 (19.9), limits choice of service facility 27 (15.8%), accident and illness not common in the HH 16 (9.4%). Almost all (98.5%) of the controls responded as they desired to renew their CBHI membership year after year. The qualitative finding authenticated that unavailability of the drug, poor service quality, and poor provider attitude affect households' sustainability in the program.

FGD discussants stated:

As [for] me, the main cause of dropout from the program is the unfeasible promise made by the government at the beginning while motivating the community to enroll in the program; they advertised that once the HH is registered and enrolled in and possesses a program card, they will get full service without additional payment, but in our contractual facility we couldn't get any: no laboratory, no drug (even anti-pain) so we have incurred double expense” and health providers consider the visit to the facility as being without a reason” [FGD discussant, 45 years old male, kebele administrator].

Other FGD discussants strengthen the above idea as:

Our catchment community perceives a health service provider does not treat CBHI members the same as direct payers. Previously, some providers have considered CBHI members to frequently visit health facilities even when they are not in real need, but such issues have been resolved through discussion. Even though we are striving to do our best to solve the existing problems, still there are challenges in the drug availability. “And some patients in chronic care follow-up who are referred to Atat Hospital complain that they must have a referral for every visit and that is uncomfortable. [Male, 29 years old, health center head].

### Another CBHI dropout FGD discussant pointed out

As we can't get the ordered drug, how and why would we be interested to renew the membership card? In addition, the package does not cover older parents under my responsibility, so I decided to stop; why [should] I pay in two ways? [Male, 47 years old].

About 47.4% of cases and 81.9% of controls reported that there had been an illness in a family member in the last 3 months. About 22.2% of cases and only 0.7% of controls reporting sick HH members did not visit a health facility for their illness. Their main reason was they had no money 52.6%, the facility was too far away 31.6%, and not getting quality service during prior visits 68.4% (multiple responses).

### Factors influencing dropout from community based health insurance

A total of 33 variables were considered for bivariate analysis, 21 of them had a *p*-value < 0.2 hence were selected and tested for independence by multivariable analysis. A backward stepwise LR (likelihood ratio) logistic regression method was used; in the final model, there were 10 variables independently associated (*p* < 0.05) with dropout from CBHIS. Accordingly, the odds of being a dropout from CBHI among HHS belonging to the wealthiest economic status (4th and 5th quintiles) were more likely compared to households of the poorest wealth status (AOR: 2.36, 95% CI: 1.14–4.87) (Table 3). The qualitative finding strengthens the survey finding.

**Table 3:** Factors influencing dropout of HH from CBHI membership adjusted for confounding variables, in rural districts of Gurage Zone, Southwest Ethiopia,2021 (*n* = 513).

**Variables**	**Category**	**Drop out from CBHI**		**COR (95%C.I)**	**AOR(95%C.I)**	**P-value**
		**Yes (%)**	**No (%)**			
Households' wealth status (in quintiles)	Lowest quintiles	69(40.6)	136(39.6)	1.00	1.00	
	Middle quintiles	44(25.6)	60(17.6)	1.43(.97–2.24)	1.68(.87–3.48)	
	Wealthiest quintiles	58(33.8)	146(42.8)	0.76(.48–1.20)	2.36(1.14–4.87)*	≤ 0.02
Attitude toward CBHI	Favorable	93(54.4)	281(82.2)	1.00	1.00	
	Unfavorable	78(45.6)	61(17.8)	3.86(2.56-5.81)	1.81(1.87–3.37)*	≤ 0.05
Illness experience in the last 3 months	Yes	74(43.3)	279(81.6)	1.00	1.00	
	No	97(56.6)	63(18.4)	5.80(3.81–8.72) *	5.21(2.90–9.33)*	≤ 0.001
Exposure to health facility	>5 times/yr	149(87.1)	339(99.1)	1.00	1.00	
	≤ 5 times /year	22(12.8)	3(0.9)	16.68(4.91–56.6)*	5.03(1.17–23.40)*	≤ 0.030
Indigenous community insurance member	Yes	131 (76.6)	309 (90.4)	1.00	1.00	
	No	40 (23.4)	33 (9.6)	2.85(1.72–4.73)*	0.10(0.03–0.37)*	≤ 0.001
graduated MHH	Yes	78(45.6)	231(67.5)	1.00	1.00	
	No	93(54.4)	111(22.5)	2.40(1.7–3.60) *	3.20(1.75–5.83) *	≤ 0.001
Duration of Membership	>3yrs	63(36.8)	224(65.5)	0.35(.21–0.45) *	0.55(.29–0.94) *	≤ 0.050
	< =3yrs	108(63.2)	118(34.5)	1.00	1.00	
Trust in the CBHI committee	Yes	82 (48.0)	320 (93.6)	1.00	1.00	
	No	89(52.0)	22(6.4)	15.78(9.3-26.73) *	10 (4.70-23.53) *	≤ 0.001
Ordered drug available	Yes	91(65.0)	330(96.5)	1.00	1.00	
	No	49(30.0)	12(3.5)	14.8(7.55–29.01)*	14.60(5.37–39.83)*	≤ 0.001
Waiting time	>3 h	30(21.4)	23(6.7)	3.78(2.10–6.78)*	4.20(1.70–10.66)*	≤ 0.002
	≤ 3 h	110(78.6)	319(93.3)	1.00	1.00	
Perceived service quality	Good	2 (1.4)	85 (24.8)	1.00	1.00	
	Poor	138(98.6)	257(75.2)	22.8(5.53–94.15) *	12.3(2.46–62.24)*	≤ 0.002

*Statistically significant.

AOR, adjusted odds ratio; CBHI, community-based health insurance; COR, crude odds ratio; CI, confidence interval.

An FGD participant health extension worker explained: In my ward, those households who have children abroad and those households with a better economic status mostly choose to drop out of CBHI by opting for service from a private provider and choose out-of-pocket payment considering they get better service [Female, 32 years old].

The finding of this study also showed that respondents' attitudes toward CBHI had a strong association to dropout in CBHI, HHS with unfavorable attitudes toward CBHI were nearly two times more likely to drop out than those who had favorable attitudes (AOR:1.81, 95% CI:1.87–3.37) (Table 3). The qualitative findings authenticated how the attitude of the households toward CBHI affects program renewal. One of the CBHI member FGD discussants explained:

As I said, if implemented as intended CBHI is among the best new initiatives set by the government, because the annual expense can't cover one person's /one family member/ yearly medical cost, but we can use it for the whole family even with the existing several challenges (even though we can't get the service as the government promised to provide) [Male, 45 years old].

Contrary to the above, a dropout FGD discussant from Cheha district elaborated on the issue as follows” even the basic concept is acceptable, in my community not only me all the community members have an unfavorable attitude toward CBHI due to the challenge in the contractual health facility, besides unavailability of the promised benefit package, health professionals do not handle (treat) CBHI members as those who pay out of pocket. Unless those members do not get the chance to see the situation of the contractual facility, no one renews his program card [Male, 42 years old].

Correspondingly, illness experience in the HH showed a strong association with CBHI dropout rates. HH with no illness experience in the last 3 months were nearly five times more likely to drop out (AOR:5.21, 95% CI:2.90–9.33) (Table 3). Likewise, the finding showed that frequent exposure of household members to health facilities had a strong association with dropout rates, HHS who didn't have the experience of frequent health facility visits were five times more likely to drop than their counterparts (AOR:5.03,95% CI:1.17–23.43) (Table 3). In this study, the membership status HHS in the indigenous community (IDIR and IQUB) has a negative association with dropout; the odds of dropout among HHS who were not a member of any of the indigenous community insurance were decreased by 90% as opposed to those who are a member. The qualitative finding substantiated that being a member of an indigenous community insurance may affect the sustainability of CBHI

A ward administrator who participated in FGD explained:

In our community we have various cooperative systems when a HH confronts a financial challenge to seeking medical care, for example, we have IDIR (indigenous death insurance) so during an emergency, an IDIR member can borrow money from the IDIR and reimburse it later if the patient has been cured, and if the patient has died the amount he borrowed is reimbursed from the amount he gets from the death insurance. [Male, 45 yrs old].

Another health development army leader from the same district who participated in FGD supported his idea:

In our locality, women's dinner IDIR lent money to HHS when they faced financial difficulty to seek medical care during an emergency and they reimburse it later. [Female, 56 years old]. A 45 year old dropout mother from another district strengthens others' ideas.

The finding of this study also showed that being a model HH (graduating as a model in a rural health extension program) is strongly associated with HHS dropout status, those HHS not graduated as a model were three times more likely to drop out from their CBHI program compared to those who are graduated (AOR: 3.20, 95% CI:1.75–5.83) (Table 3). Another finding from this study was when a member remained in the program for more than 3 years, it lessens the likelihood of dropout by 45% over those households who stayed as a member for ≤ 3 years. Likewise, not trusting the CBHI committee (governing bodies) increases the dropout rate from CBHI among rural HHS becoming ten times more likely compared to HHS who trust (AOR:10.52,95% CI:4.70–23.53) (Table 3). The qualitative finding also shows that being graduated as a model is helpful for HH to renew their program card. A HEW from Ezza district that participated in an FGD stated:

In my ward, households who graduated as models renewed their program cards timely and try to motivate their neighbors to do so. [Female, 32 yrs old]. A 47 year old male participant from Cheha district stated: As I observed in my area, most of the model households remain in the program longer.

In addition, our finding showed that availability of the ordered drug in the contractual facility is strongly correlated with dropout, HHS who reported the ordered drug not available are fourteen times more likely to drop out than those HHS who reported the ordered drug available (AOR:14.62,95% CI:5.37–39.83) (Table 3). Similarly, waiting time to get the service has an association with dropout, respondents who reported a wait time >3 h were about four times more likely to drop out than those who reported wait times ≤ 3 h (AOR:4.26, 95% CI:1.70–10.66) (Table 3). Last, households who perceived the quality of service they would get from a contractual health facility as poor were 12 times more liable to drop out from CBHI than those who perceived the service quality as good (AOR:12.38, 95%CI: 2.46–62.24) (Table 3). The qualitative findings substantiated how the service quality issue affects HHS to drop out of the program. One of the FGD discussants elaborated:

To tell the truth, most of the community members in my village including me use a private health facility not because there are better-qualified professionals there but because we get proper service (laboratory service, drugs, respect) even though every service is provided by one provider, but the provider might be the one who works in the government facility or at equal level, in the contractual facility I think there is a better set up but we can't get the promised service, so the government should give the necessary attention to this issue [Male, 48 years old].

## Discussion

CBHI is very crucial in addressing the quality and equitable healthcare access-related needs of the rural poor regardless of their economic status (20). However, the dropout of members from CBHI is becoming a grave challenge. Hence, this study aimed to assess factors influencing dropout from the CBHI program and compare the various characteristics among CBHI dropout and member households.

Accordingly, in our analysis, the wealthiest HHS (4th and 5th quintiles) were two times more likely to drop out compared to households of middle and poorest wealth status. This might be because as peoples' wealth status improves they might use more goods and services and also health services further than that of the CBHI service package. And might want to purchase freely as soon as possible from private healthcare service providers (they could be able to pay out of pocket). However, a study from Ghana and Uganda indicated an opposite finding that the wealthiest households were more liable to join and adhere (17, 21). The disparity might be due to differences in understanding among communities of these countries about CBHI benefits, whatever their wealth status is.

The finding of this study also reported that respondents' attitudes toward CBHI had an association with dropout; households with unfavorable attitudes toward CBHI were nearly two times more likely to drop out than those who had favorable attitudes. This is similar to study findings from Benin (22), Ghana (23), and Ethiopia (24). This could be due to inequity, i.e., if members do not get equal service with equal respect as that of fee payers due to poor provider attitude (disapproval, impoliteness, not treated equally as that of direct payers), thus putting their sustainability in the program in question, by sorting another risk coping mechanism.

Another finding from this study was that HHS with no illness experience in the last 3 months were nearly five times more liable to drop out. The finding is more or less analogous to study finding from Bangladesh (25). That might be due to people who experience illness more insisting on insurance. Likewise, the finding showed that frequent exposure of household members to health facilities has a strong association with dropout likelihood, households who didn't have experience of frequent health facility visits were five times more liable to drop out than their counterparts. This might be because those who visit health facilities frequently may understand the financial catastrophe they face and prefer to remain in membership.

In this study, the likelihood of dropout among households who were not a member of any of the indigenous community insurance (IDIR and IQUB) was decreased by 90% compared to those who are members. The possible explanation might be households who are a member of the indigenous community insurance may consider it as a guarantee to borrow money during unpredictable illness, even if they reimburse it later, so they may choose not to remain CBHI members.

Another compelling finding of this study is that not being a model HH is positively associated with dropout from CBHIS. The analysis illustrated being a model HH (graduated as a model in a rural health extension program) is strongly associated with HHS dropout status, those HHS not graduated as a model were three times more likely to drop out in comparison to those who graduated. The potential explanation for the association might be the fact that model HHS might have a better understanding and trust through their exposure to different local meetings and proximity to the chain of government structure than non-models.

Another finding from this study was the impact the length of time members stayed in the program had on the dropout rate. Members who remained in the program for more than 3 years lessened the probability of dropout by 45% compared to those households that stayed as members for ≤ 3 years. This finding was similar to the study findings from India (26) and Ethiopia (27, 28) which showed that length of enrollment has a strong association with dropout from CBHI; as they remained enrolled longer, the probability of dropout becomes less. Likewise, this evidence was augmented by a study conducted in the Dera district, northwest Ethiopia, which showed that for those who stay enrolled for ≥4 years in the CBHI program, their odds of being a dropout lessen by 61% compared to those who stay enrolled ≤ 3 yrs (29). The potential explanation might be due to the feeling that as long as they stay in membership, they may appreciate the benefit and be more satisfied and that, in turn, increases their willingness to stay in the program.

Regarding household's trust in the CBHI committee (governing bodies), our study findings showed that not trusting the CBHI committee increases dropout rates from CBHI to about ten times more likely compared to HHS who trust. This finding was aligned with a study finding from Cambodia that showed, that as the trust of HHS in the program increases, the probability of dropout from the CBHI program becomes less (30). Other study findings from Ethiopia (14, 24, 31, 32) also supported this finding. A study conducted in the manna districts of Jima Ethiopia revealed HHS' trust in the CBHI board lessens the probability of dropping out by 57% (24). This may be the result if the board (committee) shows unfailing dedication to fulfill members' interests, their financial risk protection self-assurance may increase, which in turn improves their sustainability in membership.

Another finding from our study was the availability of the ordered drug in the contractual facility is strongly correlated with dropout from CBHI, households who reported ordered drugs not available are fourteen times more probable to drop out compared with those households that reported the ordered drug available. This finding is not different from evidence from a meta-analysis conducted in LAMIC and a study conducted in Ghana and Uganda which indicated that not addressing lapses in promised services affects HHS sustainability in CBHI negatively (8, 33, 34). This similarity may be a result of the resemblance of the study area (developing countries). This is due to the fact that the unavailability of the drug in the contractual facility exposed members to the extra expense of purchasing it, so they may prefer to use other risk coping mechanisms and decide to drop out of CBHI membership. Another explanation for this can also be contractual health facilities' failure to meet the promised services which in turn causes a lack of trust and results in dropout.

Correspondingly, the finding of this study revealed that wait time is associated with dropout from CBHI, respondents who reported a wait time >3 h were about four times more probable to drop out from CBHI compared to those who reported a wait time less or equal to 3 h. This might be due to their misperception of poor provider attitude related to delay even though the actual length of waiting time is impartial for both groups (member and non-members).

Last, our study finding stated that households who perceived the quality of service they would get from contractual health facility poor were 12 times more liable to drop out of CBHI than those who perceived the service quality as good. This finding is congruent with study findings from Burkina Faso and Senegal (13, 16). Likewise, evidence from a nationwide study conducted in Ethiopia reinforced this finding (15). Another study from manna districts of Jimma Zone, of Oromia Ethiopia also showed that the probability of dropout among those who perceived poor service quality was six times higher in contrast to their counterparts (24). This may be due to the actual service provider failing to match with their prior expectation based on what they were pledged, the likelihood of their satisfaction could be low and their attitude might shift to quit their membership.

### Strength

This study tried to explore the experience of the community for the reason why HHS drop out of CBHI by incorporating a qualitative method, to obtain information for action.

### Limitation

It was better to match the qualitative participants with different compositional and contextual factors to minimize the confounder. Although these are logics, there are several assumptions in the discussion part of the manuscript while comparing the findings with previous studies. In addition, desirability bias and recall bias were among the drawbacks. But this was minimized by shortening the time to < 3 months.

## Conclusion

The findings of this study illustrated various factors which positively and negatively influence households to drop out from CBHI: wealth status, attitude toward CBHI, perceived poor provider attitude toward CBHI members, illness experience in the household, the experience of frequent health facility visits, model household graduation status, trust in CBHI committee (governing bodies), availability of the drug in the contractual health facility, waiting time and perceived quality of health service from the contractual facility, exposure to any of the indigenous insurance (IDIR and/or IQUB) and length of stay in membership. We strongly recommend all responsible stakeholders to give strong attention to promoting the community and provider's attitude toward community-based health insurance, completing model household graduation, and improving the quality of service by addressing the basic quality-related areas like waiting time, and drug availability).

### Recommendations

We strongly recommend:

Kebele HEW/kebele leaders to strongly work on HHS to increase model HHS in the kebele by implementing all health extension packages and disseminating these packages by using various strategies to understand what they are and to strengthen information dissemination and communication activities continuously by utilizing the health development army to clearly understand features of CBHI to change the attitude of HHS, hence enabling the local community to be a member of CBHI and benefit from the program regardless of their wealth status, exposure to indigenous insurance, and HH health status. We recommend that district health offices and contractual facilities strive to be responsive to their clients in meeting the promised service package. We further recommend that the Gurage Zone health department make the timely assessment to monitor and evaluate the overall functionality of Woreda CBHI programs to pinpoint and solve problems before they become major issues (e.g., service quality issues). To researchers, further study is recommended especially on supplier side factors like quality of existing CBHI services and provider-client interaction where integrating qualitative methods can be helpful.

## Data availability statement

The original contributions presented in the study are included in the article/Supplementary material, further inquiries can be directed to the corresponding author/s.

## Ethics statement

The studies involving human participants were reviewed and approved by Institutional Review Board (IRB) of Wolkite University, College of Medicine and Health Science. The patients/participants provided their written informed consent to participate in this study.

## Author contributions

All authors listed have made a substantial, direct, and intellectual contribution to the work and approved it for publication.

## Funding

Budget for data collection and management was covered by Wolkite University. However, Wolkite University doesn't cover publication fee.

## Conflict of interest

The authors declare that the research was conducted in the absence of any commercial or financial relationships that could be construed as a potential conflict of interest.

## Publisher's note

All claims expressed in this article are solely those of the authors and do not necessarily represent those of their affiliated organizations, or those of the publisher, the editors and the reviewers. Any product that may be evaluated in this article, or claim that may be made by its manufacturer, is not guaranteed or endorsed by the publisher.

## Supplementary material

Supplementary S1 File English version questionnaire.
