# Supplementary material for: Factors influencing drop-out of households from community based health insurance membership in rural districts of Gurage Zone, Southern Ethiopia: Community based case-control study
Source: Front Public Health. 2022 Oct 5;10:925309. doi: 10.3389/fpubh.2022.925309 (PMC9581137; doi:10.3389/fpubh.2022.925309)
Supplement: Supplementary S1 File — English version questionnaire. [file Table_1.DOCX]

**Study information sheet**

Good morning /after noon -------------------------------

I am academic staff member in Worksite University College of medicine and health science department of public health. To perform research on **Factors Influencing Drop-out of Households from Community Based Health Insurance Membership In Rural Districts of Gurage Zone.** from November 2020 to june 2021 the information will be collected by using pretested structure questioners.

There is no direct benefit or risk for respondents, but the information obtained from this survey by your participation will help various stakeholders to design better strategy to minimize the financial hardship for health care .

You have been selected randomly for this interview .I would like to ask you afew questionif you have the right to refuse to participate, and you can end the interview at any time ,the interview will take approximately 30 min. the information we collect from you not be shown to anyone. If you have any question about this study you can ask now and contact us by using the following address Mobile:0922732973 Email: zkebebush @gmail.com

**Verbal consent form**

It has been read to me in the language I understand about the above stated conditions therefore I am willing to participate in this study.

Result of interview

1. completed 2.respondent not available 3.refuse 4.partially completed

**Checked by**

Supervisor Name --------------------signature-----------date--------/----------/-----E.c

Started hour -----------minute-----------------------

Questionnaire no--------------------------------------

Time interview ended ---------------hour ---------minute-----------

Name of interviewer-------------signature-----------------Date--------/--------/-------E.c

If respondent doesn’t agree to be interviewed .thanks her and go to the next responden

**9. Annex: Tool (it will be modified soon)**

**Section 1: Socio-demographic characteristics of Households**

Enumerator: Only the head of the household should be interviewed or in his/her absence the spouse should be interviewed.

| No | Variables | Alternative responses (circle the response |
| --- | --- | --- |
| 101 | Sex | 1.male 2.female |
| 102 | How old are you? | _____years |
| 103 | What is your status in the household? | 1. Husband 2. Wife  3. Child 4. Relatives  5.Others (specify)___ |
| 104 | What is your religion? | 1. Orthodox 2. Protestant  3. Catholic 4. Muslim  5. Wakefata 6. Others(specify)___ |
| 105 | To which ethnic group do you belong? | 1. Gurage 2.Kebena 3.Oromo  4. Amhara 5.Tigre 5.Other (specify |
| 106 | What is your Current marital status? | 1. single 2. Married  3. Divorced 4. widowed  5. Cohabit |
| 107 | What is your occupation? | 1.Farmer 2. Housewife 3. Merchant 4.Laborer 5.Student 6 Other(Specify)________ |
| 108 | What is the highest level of school you have completed? | 1. Can’t read and write (illiterate)  2. Can read and write  3. Primary  4.Secondary and above |
| 109 | How many members are there in your families (HH Size), including you? | ____in number |
| 110 | How many members are there in your family in each age group stated in the bracket including you? | 0-1 year), 2___(1-5year), 3__(5-18year) 4__(18-63year), 5__(64 and older) |

**Section 2: Household characteristics for wealth estimation**

|  |  |  |
| --- | --- | --- |

| S.no | **Questions and Filters** | | | **Response categories** | **Skip to…** |
| --- | --- | --- | --- | --- | --- |
| 201 | Does your household have the following properties? | | A.Sickle | 0. No  1. Yes |  |
|  |  |  | B.Plough | 0. No  1. Yes |  |
|  |  |  | C.Hoe | 0. No  1. Yes |  |
|  |  |  | D.Axe | 0. No  1. Yes |  |
|  |  |  | E. Livestock(for plough) | 0. No  1. Yes |  |
|  |  |  | G.Chair/stool/ | 0. No  1. Yes |  |
|  |  |  | H.Table | 0. No  1. Yes |  |
|  |  |  | I.Injera mitad | 0. No  1. Yes |  |
|  |  |  | J.Solar | 0. No  1. Yes |  |
|  |  |  | K.Kuraz | 0. No  1. Yes |  |
|  |  |  | L.Shelf | 0. No  1. Yes |  |
|  |  |  | M. Sleeping bed with mattress | 0. No  1. Yes |  |
|  |  |  | N. Mattress (spong). | 0. No  1. Yes |  |
|  |  |  | O. Functioning radio/tape | 0. No  1. Yes |  |
|  |  |  | P.Functioning television | 0. No  1. Yes |  |
|  |  |  | Q.Stove (kerosene/electric).Mobile phone | 0. No  1. Yes |  |
|  |  |  | R. Watch (hand /watch) | 0. No  1. Yes |  |
|  |  |  | S. Mobile phone | 0. No  1. Yes |  |
| 202 | What is the main source of drinking water for members of your household?  1. Surface 2. Well protected  3.wellunprotected 4.spring protected  5.spring unprotected 6..Pipe private  7.pipe public 8.Other specify____ | | | |  |
| 203 | What is the main source of water used in your household for other purposes such as cooking and hand washing?  1. Surface 2. Well protected  3.wellunprotected 4.spring protected  5.spring unprotected 6..Pipe private  7.pipe public 8.Other specify____ | | | |  |
| 204 | What is the source of light in your household?  1.Electric 2. Solar 3. Kuraz/gas 4.Wood | | | |  |
| 205 | What type of toilet your household used?   1. Has roof and wall and cleanable floor / slab / 2. Has no roof and wall and cleanable floor   Bush/Field 4. No toilet /Communal. 5. Other specify____ | | | |  |
| 206 | What type of fuel does your household mainly use for cooking?  1.Bush 2.dung 3.Wood  4. Charcoal 5. Electricity 6. Other specify_____ | | | | ______ |
| 207 | Do you have separate kitchen for cooking? | | | 0. No  1. Yes |  |
| 208 | To whom the house you are living in belongs to? | | | 1.private  2. not private |  |
| 209 | What is the main material of the floor in your house? | | | 1. Earth  2. Cement  3. Other specify |  |
| 210 | What is the main material of the wall in your house? | | | 1.Mud  2. Mud and Cement  4. Bricks  3. Other specify |  |
| 211 | What is the main material of the roof in your house? | | | 1. Thatched  2. Corrugated iron  3. Other specify________ |  |
| 212 | How many rooms in this household are used for sleeping? | | | _________ |  |
| 213 | No of person per sleeping room | | | _____________ |  |
| 214 | Does your household own agricultural land? | | | 0. No  1. Yes | If 0→Q216 |
| 214 | If yes to Q13, how many hectares? | | | ________ |  |
| 215 | If yes to Q13, by what are you cultivating? | | | 1. Hand  2. Oxen  3. Tractor  4. Other____ |  |
| 216 | Average amount of agricultural products collected in one production year in quintal (100kg) | | | 1. Teff _____  2. Corn _____  3. Coffee __  4. Chat (in birr)  5. Other specify____ |  |
| 217 | How many of the following domestic animals does your household have in number? | Cows and oxens together | | _________ |  |
|  |  | Goats and sheep together | | __________ |  |
|  |  | Chickens | | __________ |  |
|  |  | Horses, donkeys and mules together | | __________ |  |
| 218 | How much money in Birr from these sources of income does your HH earned in the past 12 months? | monthly salary if daiy laborour | | __________ |  |
|  |  | Sale of agricultural products | | __________ |  |
|  |  | Sale of livestock | | ___________ |  |
|  |  | Provision of any services including house rent, land rent, traditional healing, etc. | | ___________ |  |
|  |  | Money received from gov’t/ aid | | ___________ |  |
|  |  | Money received from somebody working/living outside Ethiopia | | ___________ |  |
|  |  | Total yearly income of the HH | | __________ |  |
| 219 | Does any member of your HH have a bank or microfinance saving account? | | | 0. No  1. Yes |  |

**Section 3:** Individual HHH level variables (**awareness and attitude** towards CBHI)

**3:1 Awareness about CBHI**

| No | \| Questions/variable \|  \| \| --- \| --- \| | Alternative responses (circle the response |  |
| --- | --- | --- | --- | --- | --- |
| 301 | Do you know about CBHI? | 1.Yes  0.No |  |
| 302 | If yes to “Q301, which one explains it best?  1.Prepayments for health care, 2. paying tax for Gov’t,  3.free health delivery by Gov’t 4.Other, specify___ | |  |
| 303 | From which one of the following you got about it?  1 Neighbors/friends 2. CBHI officials in public meeting  3. CBHI house to house awareness creation campaigns  4. Mass media: radio/Tv 5. Health professionals 6. others______ | |  |

**4: Attitude towards CBHI**

| **NO** | **Variables** | **Level of agreement** | | | | |
| --- | --- | --- | --- | --- | --- | --- |
|  |  | **Strongly agree(5)** | **Agree(4)** | **Neutral(3)** | **Disagree(2)** | **Strongly disagree(1)** |
| **401** | Community based health insurance has the potential on promoting health care seeking behavior from modern health care institutions |  |  |  |  |  |
| **402** | CBHI protects households from unaffordable healthcare expenditures. |  |  |  |  |  |
| **403** | Premium payment for CBHI scheme is expensive. |  |  |  |  |  |
| **404** | CBHI is means of collecting revenue (profit) to the government. |  |  |  |  |  |
| **405** | CBHI scheme members receive low quality of services than non  Members |  |  |  |  |  |
| **406** | Mistreatment of patients by the professionals is common for members than non- members. |  |  |  |  |  |
| **407** | I did not have trust in management and administration of CBHI scheme |  |  |  |  |  |
| **408** | CBHI is relevant only to promote health condition of the poor. |  |  |  |  |  |
| **409** | Health insurance is good to pool the risk of health expenditures within the sick and the healthy |  |  |  |  |  |
| **410** | \| Health insurance should be advocated and scaled up to improve health condition of rural community \|  \| \| --- \| --- \| |  |  |  |  |  |

| **Section 5: Households health and Health service related variables** | | | | | **Skip to** |
| --- | --- | --- | --- | --- | --- |
| **501** | How do you rate the health status of you and your family? | 1. Excellent 2. Very good 3. good 4.Faire 5.poor | | |  |
| 502 | Have you and your family fallen ill in the last 3 months? | 1.Yes  0.No | | |  |
| 503 | Have you and your family accustom to visit HF for any illness? | Yes  0.No | | |  |
| **504** | Do you or other member of the household have chronic illness or who have been on follow up treatment? | 1.Yes  0. No | | |  |
| 505 | Have you ever been member of micro group/saving used for health care cost or for any other else? | 1.Yes  0. No | | |  |
| 506 | Is your HH graduated by HEW as model family? | 1.Yes  0.No | | |  |
| 6.CBHI Scheme and Health service related factors | | | | | |
| 6.1 | **For How many yrs you/your HH stayed in membership of CBHI** | **Specify in yr-------** | | |  |
| 602 | **Is the annual premium of CBHI affordable** | 1. **Yes**   **0.No** | | |  |
| 603 | **Who covered your registration fee and annual premium** | 1. **My self** 2. **Government subsidy as the poorest group** | | |  |
| 604 | Do you trust the CBHI committee? | 1.Yes  0.No | | |  |
| 605 | What is the nearest HF to which the government made contractual agreement for members to medical care? | 1. HC(Gov) 2. HC(Mision) 3. Hospital(Gov.)4.Hospital(mission) 5. Other specify___ | | |  |
| 606 | How Long does it take to reach the contractual HF from your home? | ________minute | | |  |
| 607 | How do you perceive the transportation cost from your home to contractual HF? | 1. High 2. Fair  3.Cheap  4. No transportation access | | |  |
| 608 | Have you visited the contractual health facility for the illness felt in.? | 1.Yes  0.No | | |  |
| 609 | How long have you and/or your family Waited to get the services | 1. Less than 30 minutes  2.30 to 60 minutes  3.1to 3 hours  4.3 to 6 hours  5. 6 hours and more  6.More than a day | | |  |
| 610 | What was the availability of drugs/supplies looks like? | 1.Not available  2.Rarely available  3.Usually available  4. Always available | | |  |
| 611 | How can you rate the quality of using existing CBHI benefits packages? | 1.Excellent,  2. Satisfactory  3.Poor | | |  |
| 612 | How was your satisfaction with the health care services given or on health care providers? | 1.Very satisfied  2.Satisfied  3.Neutral  4.Dissatisfied  5.very dissatisfied | | |  |
| 613 | Is your HH currently member of CBHI scheme? | 1.Yes  0.No | | | If 0→Q 615 |
| 614 | You already dropped out, Why do you think that your HH have not renewed membership yet?  (multiple responses allowed) **For non-members(Dropped out HHS) only**  1. Illness and injury does not occur frequently in our HH  2. Annual premium not affordable  3. Drug not available in the contractual facility/Not reimbursed that we buy from outside  4. The quality of service (waiting time, staff attitude, medicine, diagnostics) for CBHI members is not as good as for out of pocket paying patients (non-CBHI members)  5. Benefit packages of CBHI does not cover all services of health care (referral, transportation etc.)  6. Restrict me from using service from HF I preferred  7. other, specify | | | |  |
| 615 | When your current membership expires, would you renew your CBHI membership for the following year? (**for members only**) | | | 1.Yes  0.No | If 1 to Q617 |
| 616 | Why do not you plan to renew your CBHI membership (**multiple responses** allowed)? (for members only)  1. Illness and injury does not occur frequently in our HH  2. Annual premium not affordable  3. Drug not available in the contractual facility/Not reimbursed that we buy from outside  4. The quality of service (waiting time, staff attitude, medicine, diagnostics) for CBHI members is not as good as for out of pocket paying patients (non-CBHI members)  5. Benefit packages of CBHI does not cover all services of health care (referral, transportation etc.)  6. Restrict me from using service from HF I preferred  7. other, specify | | | |  |
| 619 | Was there any of your family member felt in illness in the last 3 months | | 1.Yes  0.No | | If 0→ end |
| 620 | Have you or your family visited health facility for the illness felt in the last 3 month? | | 1.Yes  0.No | | If 1→ end |
| 621 | What was the major reason for not visiting the health facility?  1.did not feel it was necessary  2.facility too far  3. lack of money  4.did not feel that I would get quality care  5.other, specify | |  | |  |

**Thank you very much for your cooperation!**

Name of **interviewer/data collector** -------------- Date------------- Signature-----

Name of **supervisor-**------------- Date-------------------- Signature------------------
